# Domain Localization by Graphene Oxide in Supported Lipid Bilayers

**DOI:** 10.3390/ijms24097999

**Published:** 2023-04-28

**Authors:** Ryugo Tero, Yoshi Hagiwara, Shun Saito

**Affiliations:** Department of Applied Chemistry and Life Science, Toyohashi University of Technology, Toyohashi 441-8580, Japan

**Keywords:** lipid bilayer membrane, lipid raft, graphene oxide, fluorescence microscopy, atomic force microscopy, phase separation

## Abstract

The gel-phase domains in a binary supported lipid bilayer (SLB) comprising dioleoylphosphatidylcholine (DOPC) and dipalmitoylphosphatidylcholine (DPPC) were localized on graphene oxide (GO) deposited on a SiO_2_/Si substrate. We investigated the distribution of the gel-phase domains and the liquid crystalline (L_α_) phase regions in DOPC+DPPC-SLB on thermally oxidized SiO_2_/Si substrates with GO flakes to understand the mechanism of the domain localization on GO. Fluorescence microscopy and atomic force microscopy revealed that the gel-phase domains preferably distributed on GO flakes, whereas the fraction of the L_α_-phase increased on the bare SiO_2_ surface which was not covered with the GO flakes. The gel-phase domain was condensed on GO more effectively at the lower cooling rate. We propose that nucleation of the gel-phase domain preferentially occurred on GO, whose surface has amphiphilic property, during the gel-phase domain formation. The domains of the liquid ordered (L_o_) phase were also condensed on GO in a ternary bilayer containing cholesterol that was phase-separated to the L_o_ phase and the liquid disordered phase. Rigid domains segregates on GO during their formation process, leaving fluid components to the surrounding region of GO.

## 1. Introduction

A lipid bilayer is a self-assembled structure of amphiphilic lipid molecules, such as phosphatidylcholine (PC), sphingomyelin (SM), and cholesterol (Chol), in aqueous solution. The lipid bilayer is the fundamental structure of the cell membrane and also other biological membranes of organelles. Lateral organization of lipids and proteins in cell membranes plays key roles in the trafficking of substances and transduction of signal though the membranes [1,2,3,4]. To understand details of these processes, phase separation and domain formation have been investigated in artificial lipid bilayer systems. Properties in the hydrophobic core of lipid bilayers dominantly affect these phenomena. A typical example is the phase separations between the gel and liquid crystalline (L_α_) phases in a binary lipid bilayer consisting of a lipid with saturated acyl chains and that with unsaturated acyl chains. The regions in the gel phase and in the L_α_ phase coexist at a temperature between the phase transition temperatures (*T*_m_) of the two lipids depending on the lipid fraction and the temperature. Incorporation of Chol causes liquid–liquid phase separation between the liquid-ordered (L_o_) phase and the liquid-disordered (L_d_) phase. Interaction within the hydrophobic core of the lipid bilayer a dominant factor for the domain formation of lipids.

The supported lipid bilayer (SLB) is one of artificial lipid bilayer systems existing at the interface between a solid substrate and an aqueous solution [5,6]. SLB has high technical affinity to functional materials and solid sensors. Basic physical characters of bilayer membranes, e.g., lateral diffusion of molecules, phase transition, and phase separation, are retained in SLB owing to the approximately 1 nm thick water layer existing between the lipid bilayer and the substrate [7,8]. Even through this water layer, however, physical and chemical properties of substrate surfaces possibly affect the structure and characteristics of the lipid bilayer in SLB systems [6]. Representative examples are decoupled molecular diffusion and phase transition between upper and lower leaflets observed on mica substrates [9,10,11,12]. Microfabrication and chemical modification of the substrate surfaces are applied to patterned SLBs and SLB arrays. As well as barriers that physically divide lipid bilayers into isolated patches [13,14,15], roughness [16], curvature [17,18,19], and chemical condition [20,21,22] of substrate surfaces are effectively used for the patterning of SLB. These are also effective to localize lipid domains in phase-separated multi-component SLBs [16,19,23].

Recently, we reported formation and physical properties of SLB on graphene oxide (GO) that was deposited on a thermally oxidized Si wafer (SiO_2_/Si). GO is a chemical derivative of graphene, which is a two-dimensional carbon nanomaterial, modified with hydrophilic functional groups, e.g., hydroxy, carboxy, and carbonyl groups [24,25,26]. These oxygen functional groups make the hydrophobic pristine graphene hydrophilic, whereas a part of pristine graphene remains as small patches on a scale of nanometer [27,28]. The surface of GO shows amphiphilic manner although a GO flake as a whole is hydrophilic enough to be dispersed in water. SLB of dioleoyl-PC (DOPC) is formed on the GO flakes on the GO/SiO_2_/Si substrate by the vesicle fusion method, and the SLB is continuous between the regions on GO and a bare SiO_2_ surface [29,30,31]. In SLB consisting of DOPC and a lipid conjugated with polyethylene glycol (PEG), the domain of the PEG-tagged lipid selectively exists on the GO flakes and disappears from the bare SiO_2_ region [30,31]. In this study, we aim to clear the mechanism and versatility of the domain localization on GO occurring in SLBs. Domain distribution in a binary SLB of DOPC and dipalmitoyl-PC (DPPC) and a ternary SLB containing Chol on the GO/SiO_2_/Si substrate was investigated. The localization was a kinetic process, and rigid domains preferentially segregated on GO leaving the fluid component in SLB on the surrounding bare SiO_2_.

## 2. Results and Discussion

**Figure 1:**
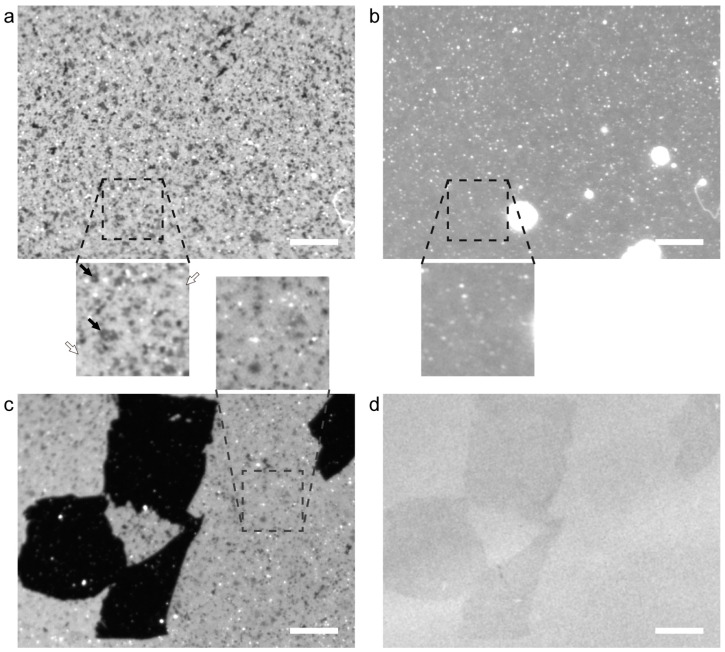
(**a,b**) Fluorescence images of DOPC+DPPC-SLB on the SiO_2_/Si substrate without GO obtained at the same position at (**a**) 25 °C and (**b**) 45 °C accompanied with the magnifications of the dotted square regions (25 × 25 µm^2^). The black and white arrows in (**a**) indicate representative gel-phase domains and the L_α_-phase regions, respectively. (**c**) A fluorescence image of DOPC+DPPC-SLB on the GO/SiO_2_/Si substrate at 25 °C accompanied with the magnifications of the dotted square region (25 × 25 µm^2^). (**d**) A bright field image obtained at the same position as (**c**). Scale bar = 20 µm.

We prepared DOPC+DPPC-SLB at 45 °C by incubating thermally oxidized SiO_2_/Si substrates in a suspension of vesicles comprising DOPC and DPPC. The transition temperatures between the gel and L_α_ phases of DOPC and DPPC are −17 °C and 41 °C, respectively. During the SLB formation at 45 °C, DOPC and DPPC was uniformly mixed. In a DOPC+DPPC bilayer at the molar fraction of 1:1, the gel phase and the L_α_ phase coexist below 30 °C as known in its phase diagram [32,33]. Figure 1a shows a fluorescence image of DOPC+DPPC-SLB on the SiO_2_/Si substrate observed at 25 °C. Small dark areas in Figure 1a (indicated by black arrows) correspond to the gel-phase domains, which mainly consist of DPPC, segregated in the L_α_-phase region, in which majority of DOPC exist, showing uniform fluorescence intensity (indicated by white arrows in Figure 1a). Fluorescent lipid probes that are tagged with a bulky dye tend to distribute in the fluid L_α_-phase rather than the packed gel phase [1,34,35,36]. White dots were unruptured vesicles [37,38]. The area fraction of the gel-phase domain (*θ*_gel_) was 14.2%. This valued is consistent with the ratio between the gel and L_α_ phases in an equimolar DPPC+DOPC bilayer at 25 °C based on the phase diagram of a DPPC+DOPC bilayer in the literature [32,33]. At 45 °C, the gel-phase domains were not observed and DOPC+DPPC-SLB showed homogeneous fluorescence intensity (Figure 1b). Note that white objects in Figure 1b were multi-layered bilayers and vesicles appearing during heating due to thermal expansion of SLB.

We casted GO on the SiO_2_/Si substrate and formed DPPC+DOPC-SLB by the vesicle fusion method as with Figure 1a. Figure 1c shows the fluorescence image of the DOPC-SLB on the GO/SiO_2_/Si at 25 °C and the bright-field image of the identical position is shown in Figure 1d. The shape of each GO flake was recognized in Figure 1c because the fluorescence from the SLB on GO is quenched by GO [29,39]. Single-layered GO flakes are visualized by bright-filed observation on a SiO_2_/Si substrate having approximately 90 nm thick SiO_2_ layer because of the interference effect [40]. The bright field image in Figure 1d shows that four GO flakes existed in the view field. In the DPPC+DOPC-SLB on the bare SiO_2_ surface, where GO flakes are not observed in Figure 1d, dark gel-phase domains are observed in a homogeneously bright L_α_-phase region like in Figure 1a. The area fraction of the gel-phase domain was, however, smaller than that in Figure 1a.

**Figure 2:**
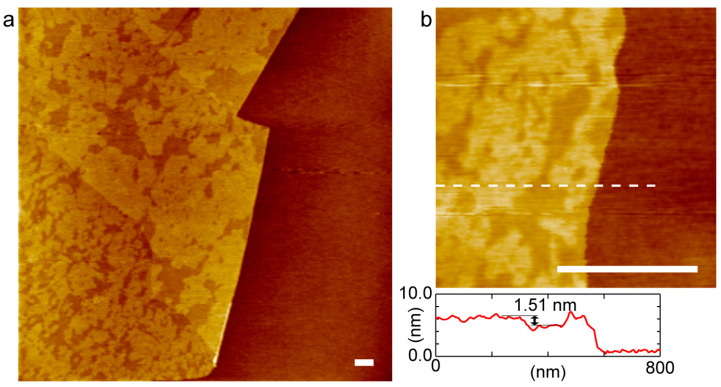
AFM topographies ((**a**) 5.0 × 5.0 µm^2^ and (**b**) 1.0 × 1.0 µm^2^) of DOPC+DPPC-SLB on the GO/SiO_2_/Si substrate at 25 °C. (**b**) The cross-section profile at the white dotted line is accompanied. Scale bar = 500 nm.

We investigated the domain distribution in DOPC+DPPC-SLB on GO by atomic force microscopy (AFM) because the fluorescence intensity on GO was too low to recognize the gel-phase domains and L_α_-region (Figure 1c). Figure 2a shows an AFM topography of DOPC+DPPC-SLB on the GO/SiO_2_/Si substrate. A GO flake existed at the left side of the image, as recognized by the height difference between the SLBs on GO and the bare SiO_2_ region by the GO thickness [29,31]. Two regions with different height existed in the SLB on GO in the left side of the image, while the SLB showed uniform height on the bare SiO_2_ region in the right side. The difference in height between the two regions in DOPC+DPPC-SLB on GO was approximately 1.5 nm (Figure 2b). It corresponds to the height difference between the gel-phase and L_α_-phase domains in previous AFM studies [34,38,41]: the former is thicker because of the all-trans acyl chains than the latter with diverse gauche conformations. The AFM topography shows that the gel phase and L_α_ phase coexisted on GO and also that the gel-phase was abundant compared to the L_α_ phase indicated by their area fractions. The homogeneous SLB on the bare SiO_2_ region is in the L_α_ phase, as indicated by the fluorescence image (Figure 1c). The height difference of 1.5 nm is larger than the difference in the thickness between the gel-phase and L_α_-phase bilayer membranes [42], mainly because a softer L_α_-phase bilayer is compressed more than a gel-phase bilayer during AFM observation in conventional intermittent contact mode. The decoupled phase separation between the upper and lower leaflets that results in three regions with different height (gel/gel, gel/L_α_ and L_α_/L_α_) [10,11] was not observed in this study.

The results in Figure 1 and Figure 2 show that the gel-phase domains preferentially located on GO. The total amount of the gel-phase domains in a whole DPPC+DOPC bilayer membrane is thermodynamically determined at arbitrary lipid component and temperature as expressed in the phase diagram [32,33]. Specific affinity between DPPC and GO seems unlikely because DPPC and DOPC have the identical phosphocholine headgroup. We surmise that the localization of the gel-phase domains on GO proceeds via kinetic processes; therefore, we investigated the dependence of the domain localization on the cooling rate.

**Figure 3:**
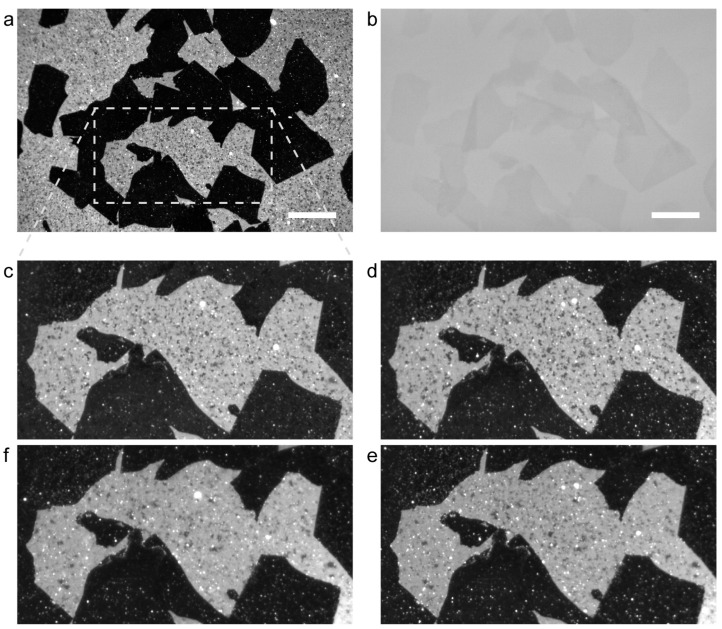
(**a**) A fluorescence image of DOPC+DPPC-SLB on a GO/SiO_2_/Si substrate. Cooling rate from 45 °C to 25 °C was 20 °C/min. (**b**) A bright field image obtained at the same position as (**a**). (**c**) The magnified image of the dotted rectangular region in (**a**). (**d–f**) Fluorescence images of the same position as (**c**) after the sample was cooled from 45 °C to 25 °C at the cooling rate of (**d**) 5.0 °C/min, (**e**) 1.0 °C/min, and (**f**) 0.5 °C/min. Note (**c**) and (**f**) are aligned vertically for feasible comparison. Scale bar = 50 µm.

Figure 3a shows fluorescence images of DPPC+DOPC-SLB on a GO/SiO_2_/Si substrate that was cooled from 45 °C to 25 °C at the rate of 20.0 °C/min. The dark regions correspond to the GO flakes that were observed in the bright field image obtained at the same position (Figure 3b). The bright regions in Figure 3a, DPPC+DOPC-SLB on the bare SiO_2_ surface, contains small dark spots that correspond to the gel-phase domains. A SiO_2_ region surrounded by GO flakes (dotted square in Figure 3a) was magnified in Figure 3c. The sample was repeatedly heated to 45 °C and cooled to 25 °C, at the cooling rate of 5.0, 1.0, and 0.5 °C/min (Figure 3d–f, respectively). The sample was kept at 45 °C for 30 min before cooling so that the components in the SLB on the GO flakes and bare SiO_2_ regions homogeneously mixed. At the cooling rate of 20 °C/min (Figure 3c), *θ*_gel_ in DPPC+DOPC-SLB at 25 °C was 6.4%. It is significantly smaller than that on the SiO_2_/Si substrate without GO (Figure 1a), 14.2%. Lower *θ*_gel_ was obtained at slower cooling rate (Figure 3d–f): *θ*_gel_ = 4.9, 3.0, and 2.4% at the cooling rate of 5.0, 1.0, and 0.5 °C/min (listed in the upper row of Table 1). On the other hand, *θ*_gel_ did not depend on the cooling rate on the SiO_2_/Si substrate without GO (the lower row of Table 1, fluorescence images are shown in Figure S1 in the Supporting Information). It is because the existence ratio in the whole bilayer is thermodynamically determined, although the shape (dendric or rounded) or size of each domain may be affected by kinetic processes [43,44]. The results summarized in Table 1 support that the domain localization on GO is kinetically induced during the domain formation.

**Figure 4:**
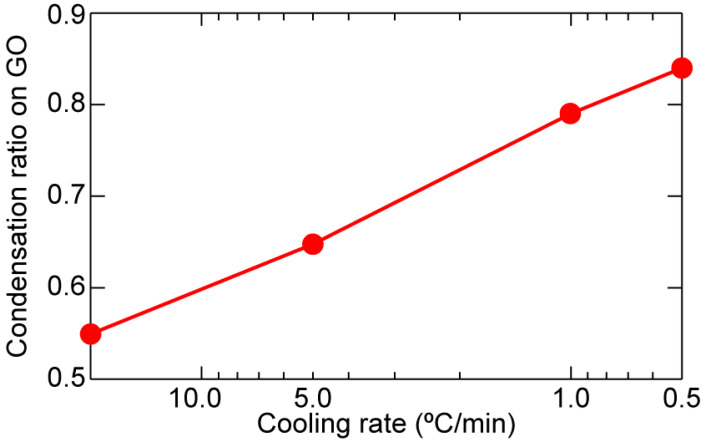
Dependence of the condensation ratio (*C*) on the cooling rate. Each value of *C* was calculated by Equation (1) using the values in Table 1.

Assuming the total amount of the gel-phase domain in the whole SLB is independent of the cooling rate and the existence of GO, we evaluate the condensation ratio (*C*) of the gel-phase domain on GO based on *θ*_gel_ values in the bare SiO_2_ region as follows:

(1) $$C=\frac{\theta_{0}-\theta_{{\mathrm{SiO}}_{2}}}{\theta_{0}}$$

where *θ*_0_ represents the thermodynamically determined area fraction of the gel-phase domains that is experimentally obtained from *θ*_gel_ on SiO_2_/Si without GO as *θ*_0_ = 14.2 (%), and *θ*_SiO2_ represents the *θ*_gel_ value of the bare SiO_2_ region on the GO/SiO_2_/Si substrate. The value of *C* at each cooling was calculated using *θ*_SiO2_ listed in the upper row of Table 1. We regard *C* as a fraction of the gel-phase domains condensed to GO. At the cooling rate of 20 °C/min (*θ*_SiO2_ = 6.4, *θ*_0_ = 14.2), *C* = 0.55 indicating that 55% of the gel-phase domains on the bare SiO_2_ region moved to GO. The dependence of *C* on the cooling rate was plotted in Figure 4. The value of *C* increased with the reductant of the cooling rate and reached 0.84 at 0.5 °C/min. GO flakes condensate the gel-phase domain in DPPC+DOPC-SLB on them, more effectively during the slower cooling.

**Figure 5:**
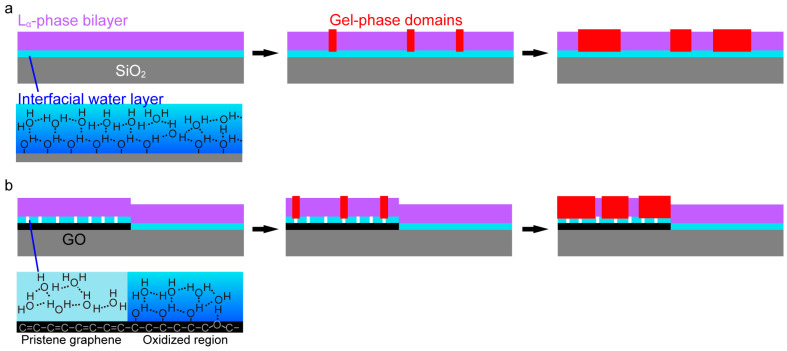
Schematics of gel-phase domain formation in L_α_-phase SLBs on (**a**) a SiO_2_/Si substrate without GO and (**b**) a SiO_2_/Si substrate with GO.

We consider the mechanism of the localization of the gel-phase domain on GO. DOPC and DPPC have the same headgroup structure; therefore, in the uniformly mixed SLB in the L_α_ phase, DOPC and DPPC distribute equally on the GO and the bare SiO_2_ regions. The domain localization occurs during the formation process of the gel-phase domains. The domain formation in a lipid bilayer proceeds via fundamental processes of crystal growth represented by the nucleation and domain growth [43,44]. During the cooling process, nucleation of the gel-phase domain occurs in a DOPC+DPPC bilayer in the L_α_ phase. In the supersaturation condition just under the transition temperature, DPPC molecules gather forming a cluster. The cluster grows to a larger domain if it becomes larger than the critical nucleus. The nucleation occurs anywhere on a homogeneous surface, while it may proceed preferentially at a specific site on a heterogeneous surface (Figure 5).

The surface of the piranha cleaned SiO_2_/Si substrate is uniformly hydrophilic because of the surface hydroxy group that forms network of water molecules through the hydrogen bond in the vicinity of the SiO_2_ surface (Figure 5a). This interfacial water layer induces “hydration repulsion”. On homogeneously hydrophilic surface, nucleation of DPPC occurs anywhere, and thus the gel-phase domain distributes randomly (Figure 5a), as shown in Figure 1a. The hydration repulsion between the substrate surface and a lipid bilayer is attenuated with the reduction in the density of the surface hydroxy group [22]. GO has a heterogeneous surface on a scale of nanometer [27,28], consisting of hydrophobic pristine graphene region and hydrophilic region modified with oxygen functional groups. A hydrophobic surface in an aqueous solution causes attraction in contrast to the hydration repulsion. Therefore, the GO surface induces laterally heterogeneous interaction: hydrophobic attraction at the pristine graphene region, and hydrophilic repulsion at the region with oxygen functional groups (Figure 5b). The cluster of DPPC, whose mobility is lower than a DOPC single molecule, is captured at the hydrophobic site on GO rather than on the homogeneously hydrophilic SiO_2_ surface at the initial stage of the gel-phase domain formation. Once the domain starts growing on GO, concentration of DPPC decreases in the SiO_2_ region resulting in the reduction in frequency of nucleation (Figure 5b). At slower cooling rate, more DPPC is provided to the growing domain on GO from the SiO_2_ region through the lateral diffusion. The dependence of *θ*_gel_ (Table 1) and *C* (Figure 4) on the cooling rate is consistent with this preferable nucleation on GO.

**Figure 6:**
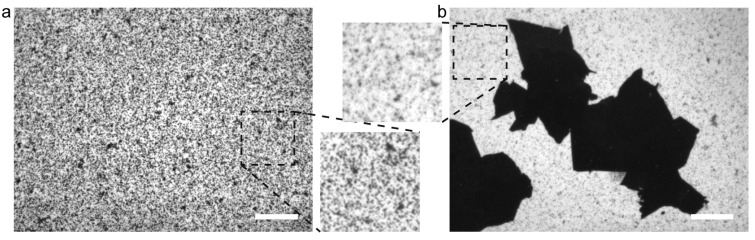
Fluorescence images of eggPC+eggSM+Chol-SLB on (**a**) the SiO_2_/Si substrate without GO and (**b**) the GO/SiO_2_/Si substrate at 25 °C accompanied with the magnifications of the dotted square regions (25 × 25 µm^2^). Scale bar = 20 µm.

Assuming this model, we predict that rigid domains segregating in a fluid phase localize on GO. We formed an equimolar ternary SLB comprising egg-derived PC (eggPC), egg-derived SM (eggSM), and Chol. Equimolar ternary bilayers comprising PC, SM, and Chol has been adopted as a representative model system of lipid rafts [1,2]. The eggPC+eggSM+Chol bilayer causes the liquid-liquid phase separation [1,2]: the L_o_ phase is rich in eggSM and Chol, and the L_d_ phase contains majority of eggPC. The former has higher viscoelasticity than the latter because Chol orders the acyl chains, while the L_d_ phase shows similar physical properties to the L_α_ phase [41]. In fluorescence observation, fluorescent lipid probes tagged with a bulky dye tend to distribute in the relatively fluid L_d_-phase rather than the viscous L_o_ phase [1,15,19,35]. In a fluorescence image of eggPC+eggSM+Chol-SLB on the SiO_2_/Si substrate (Figure 6a), the dark and bright regions correspond to the L_o_-phase domain and the L_d_-phase region, respectively. Figure 6b shows a fluorescence image of the eggPC+eggSM+Chol-SLB on the GO/SiO_2_/Si substrate. The GO flakes were observed dark as with Figure 1c and Figure 3a. The bright region corresponding to eggPC+eggSM+Chol-SLB on the bare SiO_2_ surface contained dark L_o_-phase domains, but their area fraction was smaller compared to that in the absence of GO (Figure 6a).

The phase behavior of the ternary bilayers is also expressed by phase diagrams [1,2,33]; therefore, the whole area fraction of the L_o_-phase in the equimolar eggPC+eggSM+Chol-SLB at 25 °C is determined thermodynamically. It is experimentally observed on SiO_2_/Si without GO (Figure 6a). On the GO/SiO_2_/Si in Figure 6b, decrease in the L_o_-phase domains in the bare SiO_2_ region indicates that the L_o_-phase domains preferably existed on GO. The domain localization on GO also occurred in a SLB separating to the L_o_- and L_d_-phases. Note that SM also has the same phosphocholine headgroup as PC. This result enhances that the rigid domains are condensed on GO during the cooling process as shown in Figure 5.

The molecular assembly via the hydrophobic part of lipids is the dominant factor in the model in Figure 5. However, the domain localization by GO also appear in a domain formed via the hydrophilic part of lipids. In DOPC-SLB, PEG-conjugated lipids form rigid domains, in which the lateral diffusion is hindered [45], through the intermolecular interaction between the PEG chain. The domain of the PEG-conjugated lipid localizes on GO leaving fluid DOPC-rich bilayer in the surrounding bare SiO_2_ region [31]. It is consistent with the present study that the rigid lipid domains preferably existed on GO. Additionally, flexible hydrophilic polymers induce repulsion due to the fluctuation of the polymer chains [46]. Higher repulsion is applied to an isolated PEG-conjugated lipid compared to DOPC, because of the fluctuation repulsion in addition to the hydration repulsion from the substrate. Therefore, density of the PEG-conjugated lipid is possibly higher on GO than on the bare SiO_2_ surface even in the uniform L_α_-phase.

The ratio between the hydrophilic and hydrophobic parts in GO depends on the oxygen content in GO. Oxidation degree of GO varies the assembly of lipid molecules on GO and reduced GO [47], hence possibly affects efficiency of the domain localization in SLB. The chemical property of the substrate and the interfacial water layer below GO may be another factor influencing to SLB above GO. Yamazaki et al. reported that hydrophilicity of the support substrate under graphene permeably determine the adsorption of lipid membranes on the upper side graphene [48]. Surface modification of the SiO_2_ surface in prior to GO deposition may be available for controlling the efficiency of the domain localization in SLB on GO.

In conclusion, we revealed that gel-phase domain and the L_o_-phase domain were localized on GO in DOPC+DPPC-SLB and eggPC+eggSM+Chol-SLB, respectively. The condensation of the rigid domains on GO is expressed as the preferential nucleation of GO due to the amphiphilic property of the GO surface in contrast to the uniformly hydrophilic SiO_2_ surface. These results provide fundamental information about the effect of nanocarbon materials on the biomembranes of cells and organelle. Additionally, an abundance of studies hasbeen performed for the patterning of GO on device materials and sensors [49,50,51,52]. The domain localization phenomenon is applicable to the patterning of lipid domains applying the GO-pattered substrates.

## 3. Materials and Methods

An aqueous suspension of graphene oxide was prepared through the chemical exfoliation of graphite by the modified Hummer’s method [53,54]. Briefly, graphite particles (Ito Graphite Co., Ltd., Kuwana, Japan) were oxidized in two steps with peroxydisulfuric acid and potassium permanganate in sulfuric acid. Single-layered GO flakes were obtained after the oxidized graphite was dispersed into pure water. Residual oxidized graphite particles and multi-layered GO flakes were removed by centrifugation. The GO suspension was sonicated for 30 min to reduce the size of the flakes [47]. A thermally oxidized SiO_2_/Si substrate with a 90 nm thick SiO_2_ layer was cleaned with a piranha solution (1:3 *v/v* solution of 30% H_2_O_2_ and sulfuric acid) at 180 °C for 30 min followed by sonication in 0.02 M KOH aqueous solution for 10 min. The GO suspension was cast on the SiO_2_/Si substrate and dried with an argon stream. The details of the preparation of GO and the SiO_2_/Si substrate are described elsewhere [47].

1,2-dioleoyl-*sn*-glycero-3-phosphocholine (DOPC), 1,2-dipalmitoyl-*sn*-glycero-3-phosphocholine (DPPC), L-α-phosphatidylcholine (Egg, Chicken) (eggPC), sphingomyelin (Egg, Chicken) (eggSM), and a dye-labeled lipid for fluorescence observation (1,2-dipalmitoyl-*sn*-glycero-3-phosphoethanolamine-N-(lissamine rhodamine B sulfonyl) (Rb-DPPE, Ex/Em: 560/583 nm)) were purchased from Avanti Polar Lipids, Inc. (Alabaster, AL, USA) and were used as received without further purification. The chloroform solutions of DOPC, DPPC, and Rb-DPPE were mixed in a molar ratio of 50:50:0.2 in a glass vial. Those of eggPC, eggSM, and Chol were mixed in a molar ratio of 33:33:33:0.2 in a glass vial. After the solution was dried with a nitrogen flow, the glass vial was stored under vacuum for at least 6 h. After addition of a buffer solution (100 mM KCl, 25 mM HEPES/NaOH pH 7.4) to the vacuum-dried lipid film, a unilamellar vesicle suspension was prepared through the processes of agitation, freeze–thaw cycles, and extrusion through 800 nm pore and 100 nm pore polycarbonate filters. The SiO_2_/Si substrates with and without GO were incubated in the vesicle suspension with a lipid concentration of 0.05 mM in the presence of 5 mM Ca^2+^ at 45 °C for 60 min to form SLB. The vesicle suspension was exchanged with the buffer solution after the incubation to remove excess vesicles from the aqueous phase. The details about the SLB formation by the vesicle fusion method are described elsewhere [29].

Fluorescence and bright-field observations were performed with an epifluorescence microscope (BX51WI, Olympus, Tokyo, Japan) equipped with a 60× water-immersion objective (NA = 1.00) and a CMOS camera (DS-Qi2, Nikon Solutions Co., Ltd., Tokyo, Japan). A mirror unit U-MWIG3 (Ex: 530–550 nm, Em > 575 nm, Olympus) was used for the fluorescence observation. The temperature of the SLB samples were controlled using a Peltier stage during the fluorescence observation in the range of 25–45 °C. The heating and cooling rates were 20 °C/min if not mentioned otherwise. The sample was kept at 45 °C for 30 min before cooling when the dependence of *θ*_gel_ on the cooling rate was investigated at the same position. The area fraction of domains in SLB was analyzed with an image processing software (Image J, NIH, http://imagej.nih.gov/ij/, (accessed on 25 December 2022)) by thresholding the dark gel-phase domains and the bright L_α_-phase region. Fluorescence images from at least five different positions of a sample were analyzed to calculate *θ*_gel_ that was the weighted average on the analyzed area. AFM topographies were obtained with PicoPlus 5500 (Keysight Technologies, Inc., Santa Rosa, CA, USA) using a Si cantilever (OMCL-AC240TN, Olympus, spring constant 2 N/m) in the acoustic AC mode in the buffer solution at 25 °C.

## Figures and Tables

**Table 1:** The area fraction of the gel-phase domains (*θ*_gel_) in DOPC+DPPC-SLB cooled from 45 °C to 25 °C at various cooling rates on the GO/SiO_2_/Si substrate and the SiO_2_/Si substrate without GO.

Cooling Rate (°C/min)	20.0	5.0	1.0	0.5
GO/SiO_2_/Si (%)	6.4	4.9	3.0	2.4
SiO_2_/Si without GO (%)	14.2	13.9	14.3	15.0

## Data Availability

Not applicable.

## Supplementary Materials

The following supporting information can be downloaded at: https://www.mdpi.com/article/10.3390/ijms24097999/s1.
