# Supplementary material for: Domain Localization by Graphene Oxide in Supported Lipid Bilayers
Source: Int J Mol Sci. 2023 Apr 28;24(9):7999. doi: 10.3390/ijms24097999 (PMC10178265; doi:10.3390/ijms24097999)
Supplement: Supplementary file 1 [file ijms-24-07999-s001.zip › ijms-2166518-supplementary.pdf]

Supporting Information

Domain Localization by Graphene Oxide in Supported Lipid Bilayers

Ryugo Tero *, Yoshi Hagiwara and Shun Saito

Department of Applied Chemistry and Life Science, Toyohashi University of Technology, Toyohashi 441-8580, Japan

* Correspondence: tero@tut.jp

Fluorescence images of DOPC+DPPC-SLB on SiO_2/Si prepared with various cooling rate

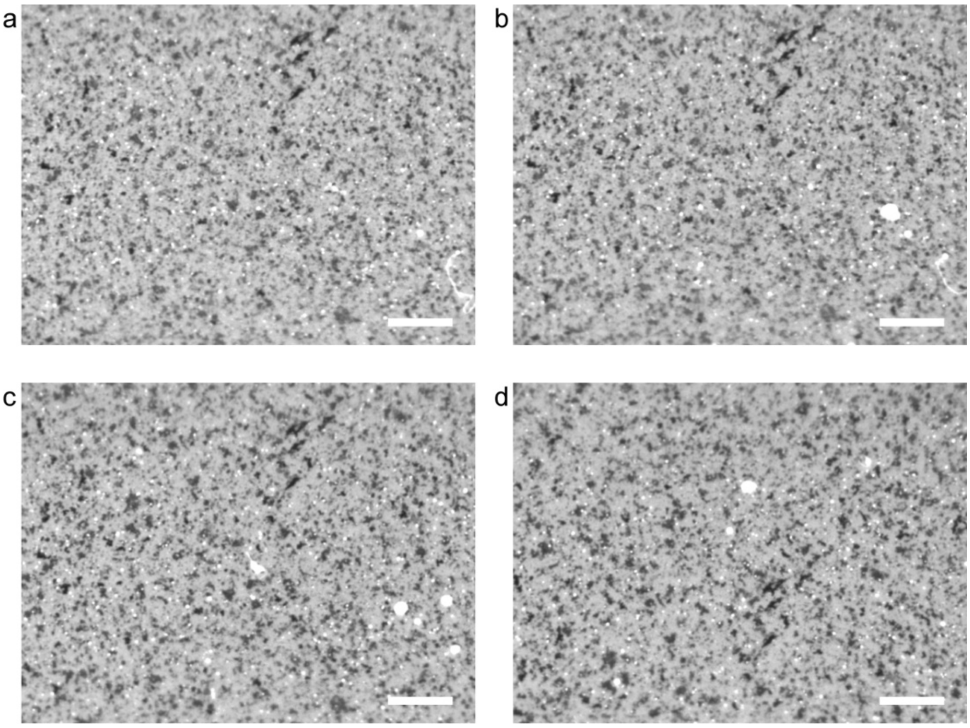

Figure S1. Fluorescence images of DOPC+DPPC-SLB on the SiO_2/Si substrate without GO cooled from 45 °C to 25 °C at various colling rates: (**a**) 20.0 °C/min (corresponding to Figure 1a in the main text), (**b**) 5.0 °C/min, (**c**) 1.0 °C/min and (**d**) 0.5 °C/min. The sample was kept at 45 °C for 30 min during the cycle of heating and cooling. Scale bar = 20 μm.
